# Satellite DNA evolution in two holocentric species of *Edessa* true bugs (Hemiptera: Pentatomidae) with unusually high heterochromatin abundance

**DOI:** 10.1007/s10577-026-09809-2

**Published:** 2026-07-31

**Authors:** Letícia M. Calegari, Diogo Milani, Vanessa B. Bardella, Frederico Hickmann, Diogo C. Cabral-de-Mello

**Affiliations:** 1 Departamento de Biologia Geral E Aplicada, Instituto de Biociências/IB, UNESP—Universidade Estadual Paulista, Rio Claro, SP 13506-900 Brazil; 2 Department of Entomology and Acarology, Luiz de Queiroz College of Agriculture, (USP/ESALQ), University of São Paulo, Piracicaba, SP Brazil; 3 Department of Entomology, 240 Wooster Science Building 1680 Madison Ave, Wooster, OH USA

**Keywords:** Heterochromatin, Holocentric chromosome, RepeatExplorer, Repetitive DNA, Satellitome

## Abstract

Satellite DNAs (satDNAs) are a major component of eukaryotic genomes, playing key roles in chromosome organization, heterochromatin formation, and genome evolution. Although satellitomes have been increasingly characterized in insects, most studies focus on monocentric species, leaving holocentric systems comparatively understudied. Here, we investigate the satellitomes of two pentatomid bugs, *Edessa meditabunda* and *Edessa loxdalii*, which exhibit unusually large heterochromatin blocks for holocentric chromosomes. Using an integrative approach combining genome analysis and molecular cytogenetics, we identified 31 satDNA families shared between species. *E. loxdalii* shows higher satDNA abundance and lower sequence divergence, consistent with recent amplification and homogenization, whereas *E. meditabunda* exhibits lower abundance and higher divergence, suggesting more advanced sequence degeneration. SatDNA landscape analyses indicate distinct evolutionary trajectories, with evidence of multiple amplification waves and differential repeat turnover. Despite these differences, both species share a conserved set of satDNAs, including highly abundant families that dominate their satellitomes, supporting the library hypothesis. Compared to other Pentatomidae species with low heterochromatin content, there is no overall increase in satDNA abundance in *Edessa*, but rather amplification of specific families. Chromosomal mapping revealed that these amplified satDNAs are major components of terminal heterochromatin blocks, while less abundant families show chromosome-specific distributions, including accumulation on the Y chromosome, with signs of evolutionary differentiation between species. Overall, this study provides new insights into satellitome organization and evolution in Pentatomidae, highlighting the role of satDNAs in shaping chromosome architecture in holocentric systems.

**Supplementary Information:**

The online version contains supplementary material available at 10.1007/s10577-026-09809-2.

## Introduction

Eukaryotic genomes, in addition to single-copy genes and multigene families with multiple copies, contain abundant repetitive DNA sequences, mainly comprising tandem repeats, such as satellite DNAs (satDNAs), and dispersed elements, represented by transposons and retrotransposons (transposable elements, TEs). These elements play important roles in genome structure and function and have a recognized impact on evolutionary processes (John and Miklos 1979; López-Flores and Garrido-Ramos 2012; Biscotti et al. 2015a; Sotero-Caio et al. 2017; Cabral-de-Mello and Palacios-Gimenez 2025). SatDNAs are sequences that, in some groups, constitute a major component of the repetitive DNA fraction, as documented in the bug *Triatoma delpontei* with a huge amount of these repeats, corresponding to more than half of its genome size (Mora et al. 2023). They are commonly enriched in heterochromatic regions, particularly in centromeric and pericentromeric domains, but an increasing number of euchromatic satDNAs have been documented (e.g. Pita et al. 2017; Sproul et al. 2020; Tunjić-Cvitanić et al. 2021; Cabral-de-Mello et al. 2023; Rico-Porras et al. 2024). These sequences play important roles in genome organization and function, contributing to centromere structure and kinetochore assembly, chromosome segregation during cell division, and the establishment and maintenance of heterochromatin. In addition, satDNAs can influence gene expression through position effects and epigenetic mechanisms and may be transcribed into non-coding RNAs involved in chromatin regulation. Due to their dynamic evolution driven, for example, by processes such as unequal recombination and local amplification, satDNAs also contribute significantly to genome diversification and chromosomal evolution among species (Biscotti et al. 2015b; Kuhn 2015; Louzada et al. 2020; Shatskikh et al. 2020; Brändle et al. 2022; Ugarković et al. 2022; Liao et al. 2023).

Among insects, satDNAs have been characterized in several orders, and their study has advanced considerably in recent years with the development of computational approaches applied to sequenced genomes, allowing the characterization of the satellitome, i.e., the complete set of satDNA families present in a genome (Ruiz-Ruano et al. 2016), and revealing patterns of evolution of these repeats at the sequence level. When combined with molecular cytogenetic analyses that enable the physical mapping of satDNAs, these approaches provide important insights into the organization, distribution, and dynamics of satDNAs at the chromosomal level. Since the review on satDNAs populating insect genomes published in 2008 (Palomeque and Lorite 2008), multiple additional species have been characterized by combination of genomic and cytogenetic approaches, belonging mainly to the orders Orthoptera (Ruiz-Ruano et al. 2016; Palacios-Gimenez et al. 2017), Hemiptera (Montiel et al. 2021; Anjos et al. 2023; Mora et al. 2023), Hymenoptera (Pereira et al. 2023; Vignati et al. 2025; Teixeira et al. 2026), Coleoptera (Montiel et al. 2022; Vidal et al. 2025a; Veseljak et al. 2026), Lepidoptera (Cabral-de-Mello et al. 2021; Gasparotto et al. 2022; 2026), and Diptera (de Lima et al. 2017; Silva et al. 2019; Rossi et al. 2025), meaningfully expanding our understanding of satDNA organization, diversity, and evolution across the group.

Although satellitomes were investigated in multiple insect orders, most knowledge has been obtained from species with monocentric chromosomes, i.e., those with a localized centromere, whereas species with holocentric chromosomes, in which kinetochore activity is distributed along the entire chromosome, remain comparatively understudied (Šatović-Vukšić and Plohl 2023; Garrido-Ramos et al. 2025). This gap is particularly relevant because holocentric species provide a distinct genomic context for investigating the organization and evolution of repetitive DNAs, as in these systems, a non-located centromere can alter the relationship between repeats and centromere function. Consequently, this chromosomal architecture may lead to alternative patterns of repeat distribution and chromosomal organization, highlighting the importance of expanding studies in holocentric lineages. Hemipteran insects possess holocentric chromosomes (Melters et al. 2012; Mandrioli and Manicardi 2020) and therefore could serve as a system for exploring how satDNAs are structured and evolve in contrast to the classical monocentric condition. Pentatomidae true bug hemipterans are characterized by highly conserved karyotypes, most commonly with 2n = 14 chromosomes and an XY sex chromosomes (males). Only a few species deviate from this pattern, exhibiting reductions or increases in diploid number, as in *Dichelops* species (2n = 12) and some *Banasa* representatives (2n = 26) (revised by Rebagliati et al. 2005). Despite this overall macrostructural stability, several studies have reported certain variability in the chromosomal organization of repetitive DNAs, including multigene families such as rDNAs, histone genes, and U snDNAs (Bardella et al. 2016; Dionisio et al. 2020; Souza-Firmino et al. 2020). In addition, extensive heterochromatin reorganization has been documented in some species. For example, species of the genus *Edessa* studied to date show large blocks of heterochromatin, in contrast to the generally low heterochromatin content observed in most pentatomids (Bardella et al. 2014; Dionisio et al. 2020).

The chromosomal data available for Pentatomidae, although still scarce, indicate that, despite their overall conserved karyotypes, their genomes may be highly dynamic, warranting deeper investigation of repetitive DNAs, which contribute significantly to genome architecture and evolution. In this context, the unusual heterochromatin patterns reported for species of the genus *Edessa* make this group particularly suitable for exploring the contribution of heterochromatin-associated satDNAs to genome and chromosome organization, both in Pentatomidae and more broadly in holocentric systems. Here, we characterize the satellitomes of two *Edessa* species, *E. meditabunda* and *E. loxdalii*, using integrated chromosomal and genomic approaches, aiming to elucidate the satDNA component of their heterochromatin, providing insights into their role in heterochromatin expansion, an aspect that remains largely unexplored in insects with holocentric chromosomes.

## Materials and methods

### Animal collection and chromosome obtaining

Adult males of two *Edessa* species were collected in two distinct regions of Brazil, *E. meditabunda* in Mineiros/Goiás (six males) and *E. loxdalii* in Piracicaba/São Paulo (nine males). Testes were dissected and fixed in modified Carnoy fixative (3:1: absolute ethanol 100%: glacial acetic acid) to be used for chromosome preparation. The animal bodies, excluding the abdomens, were kept in absolute ethanol for genomic DNA (gDNA) extraction. Both materials were stored in a freezer −20 °C until use.

The chromosome preparation slides were obtained by macerating the apical region of the testis in a drop of 50% acetic acid solution and the material was spread onto glass slides at 42 °C on a heating plate. The quality of chromosome preparations was assessed using a phase-contrast microscope. Slides were selected based on the number of metaphases observed and subsequently dehydrated in an ethanol series (70%, 90%, and 100%) for 30 s each. These slides were then stored at −20 °C until they were used for Fluorescence *in situ* hybridization (FISH) experiments. For a general analysis of the karyotypes and heterochromatin organization, the slides were stained with Giemsa 5% and used in C-banding experiments according to Sumner (1972), respectively.

### Analysis of satellitome in *Edessa* species

The gDNA was extracted from the thoracic muscle tissue of one male individual from each species using the Wizard Genomic DNA Purification Kit (Promega, Madison, WI) following the manufacturer’s protocol. Genomic sequencing was performed on the Illumina HiSeq 4000 platform using the service of Macrogen Inc. (Seoul, Republic of Korea). Approximately 1 Gb of 2 × 151 bp reads were produced for each library (reads accession number: PRJNA1501319), which was used to identify the satDNA content (**accession numbers: PZ745298-PZ745328**).

For satDNA family identification, the TAREAN tool implemented within the RepeatExplorer2 pipeline (version 0.3.8–451; Novák et al. 2017; 2020) was employed exclusively for the de novo detection and classification of satDNA families from raw sequencing reads. Satellite DNA abundance was subsequently estimated using RepeatMasker Open-4.0 (Smit et al. 2015; see below for details). The analysis was performed using raw genomic reads in FASTQ format, consisting of 500,000 randomly selected paired-end reads from each sample, obtained through the Galaxy/RepeatExplorer2 utilities Preprocessing of FASTQ paired-end reads tool, including trimming, quality filtering, cutadapt filtering, and interlacing with all default parameters (Value of cutoff = 10 with 95% above cutoff). Broken pairs were discarded from the sample. All tool parameters were used according to the default RepeatExplorer2/TAREAN recommendations. SatDNA families considered in this study correspond to the satDNA identified directly by the TAREAN and subsequently selected for further characterization. To avoid redundancies between the satDNA families found at intra- and inter-species levels, a homology analysis was performed between the consensus sequences identified in each of the species and among them, through manual inspection using Geneious 4.8.5 software (http://www.geneious.com), employing multiple sequence alignments using MUSCLE (Edgar 2004). Sequences that shared at least 80% level of similarity between them were considered the same satDNA family. The tandem organization of the identified satDNAs was manually assessed using dot plot and coverage analyses in Geneious 4.8.5, and further evaluated with RepeatProfiler by mapping raw paired-end reads against a trimer of each satDNA consensus sequence (Negm et al. 2021). The use of trimers allowed us to examine read coverage across adjacent monomer junctions, where continuous coverage supports a tandem organization. After these analyses the pool of satDNAs identified in the two species were used for abundance quantification and estimation of nucleotide divergence. For satDNA families detected by TAREAN in both species a single consensus sequence was generated using all reads from both species. In addition, all sequences were checked for similarity with previously identified sequences through a comparative analysis with nucleotide sequences deposited in the Repbase/GIRI using CENSOR (Jurka et al. 1996) and NCBI databases, following threshold from Rico-Porras et al. (2025), which require a minimum monomer coverage of > 50% and sequence similarity of > 65%. Furthermore, the taxonomic group in which each sequence was formerly described was also considered to assess the potential biological significance of the matches.

The relative genomic abundance and nucleotide divergence of each sample were estimated using RepeatMasker Open-4.0 (Smit et al. 2015). To perform this analysis, raw reads from each genome were first quality-filtered, concatenated, and converted into FASTA format using the rexp_prepare_normaltag.py script (https://github.com/fjruizruano/ngsprotocols). The resulting dataset, corresponding to 1,000,000 paired-end reads per sample, was then used as input for RepeatMasker together with the satDNA consensus sequences. Sequence divergences were calculated based on Kimura 2-parameter (K2P) distances using the calcDivergenceFromAlign.pl script from the RepeatMasker package, following default recommended parameters (Smit et al. 2015). Genomic abundance for each satDNA family was calculated based on the total number of nucleotides aligned by RepeatMasker to each consensus sequence, normalized by the total size of the input read library. The satDNAs families were named as “EdeSat” considering the decreasing order of abundance estimated by mean value of abundances between the two species, followed by the nucleotide monomer length, similarly employed by Ruiz-Ruano et al. (2016). All satDNA sequences identified are available at GenBank under the accession numbers **XXXX-XXXX**.

A comparative analysis was conducted to investigate the relationship between the satellitomes of the two species. SatDNA landscapes (K2P divergence versus genomic abundance) were generated for the complete satellitomes and for the four most abundant satDNA families in each species. These landscapes were then compared to assess patterns of satDNA amplification and homogenization. Statistical correlation inferences were used to compare and understand the level of relationship of the satDNA library between the species. Based on the results of the Shapiro–Wilk normality test, we selected appropriate correlation analysis tests for normal or non-normal distribution of data with the significance threshold set at *P* < 0.05. Then, a correlation matrix using appropriate tests was employed to verify proximity of the species using genome abundance and divergence values of satDNA library of each species. All statistical tests were performed using Jamovi version 2.4.12 application (https://www.jamovi.org).

### Satellite DNA probes and Fluorescence *in situ *hybridization

Aiming to identify the chromosomal organization of certain satDNAs, we mapped them using FISH, using as probes the four most abundant families, considering the mean abundance across species. The selected satDNAs were amplified independently in each species via Polymerase Chain Reaction (PCR) using specific primers designed manually or in Geneious using the consensus sequence of each satDNA (Supplementary Table 1). Each PCR was performed using a mixture of 10 × PCR Rxn Buffer, 0.2 mM MgCl2, 0.16 mM dNTPs, 2 mM of each primer, 1 U of Taq Platinum DNA Polymerase (Invitrogen), and 50–100 ng/μL of template DNA. The template DNA was from the same individual used for genome sequencing. PCR conditions included an initial denaturation at 94 °C for 5 min and 30 cycles at 94 °C (30 s), 55 °C (30 s), and 72 °C (80 s), with a final extension at 72 °C for 5 min. The PCR products were evaluated by a 1% electrophoretic agarose gel considering that the fragments showed the expected size from the satDNA monomer lengths. All fragments were individually excised from the gel, purified, and used as templates for PCR reamplification with the same primers and conditions. The probes were labeled with biotin-14-dATP or digoxigenin-11-dUTP by Nick Translation, using the product from the second PCR as a template.

FISH experiments were performed according to Cabral-de-Mello and Marec (2021). Probes labeled with digoxigenin-11-dUTP were detected using anti-digoxigenin rhodamine (Roche), whereas the biotin-14-dATP labeled probes were detected using streptavidin, Alexa Fluor 488 conjugate (Invitrogen, Carlsbad, CA). The chromosomes were counterstained with DAPI (4′,6-diamidino-2-phenylindole, dihydrochloride; Sigma-Aldrich), and the slides were mounted with VECTASHIELD (Vector, Burlingame, CA, USA). The chromosomes and hybridization signals were observed using an Olympus microscope BX61. Fluorescence images were recorded using a DP71-cooled digital camera in greyscale. At least 15 cells per experiment were photographed, aiming at the precise description of the chromosomal location patterns. Images were pseudo-colored, merged (chromosomes and probe signals), and optimized for brightness and contrast in Adobe Photoshop CS6.

## Results

**Fig. 1:**
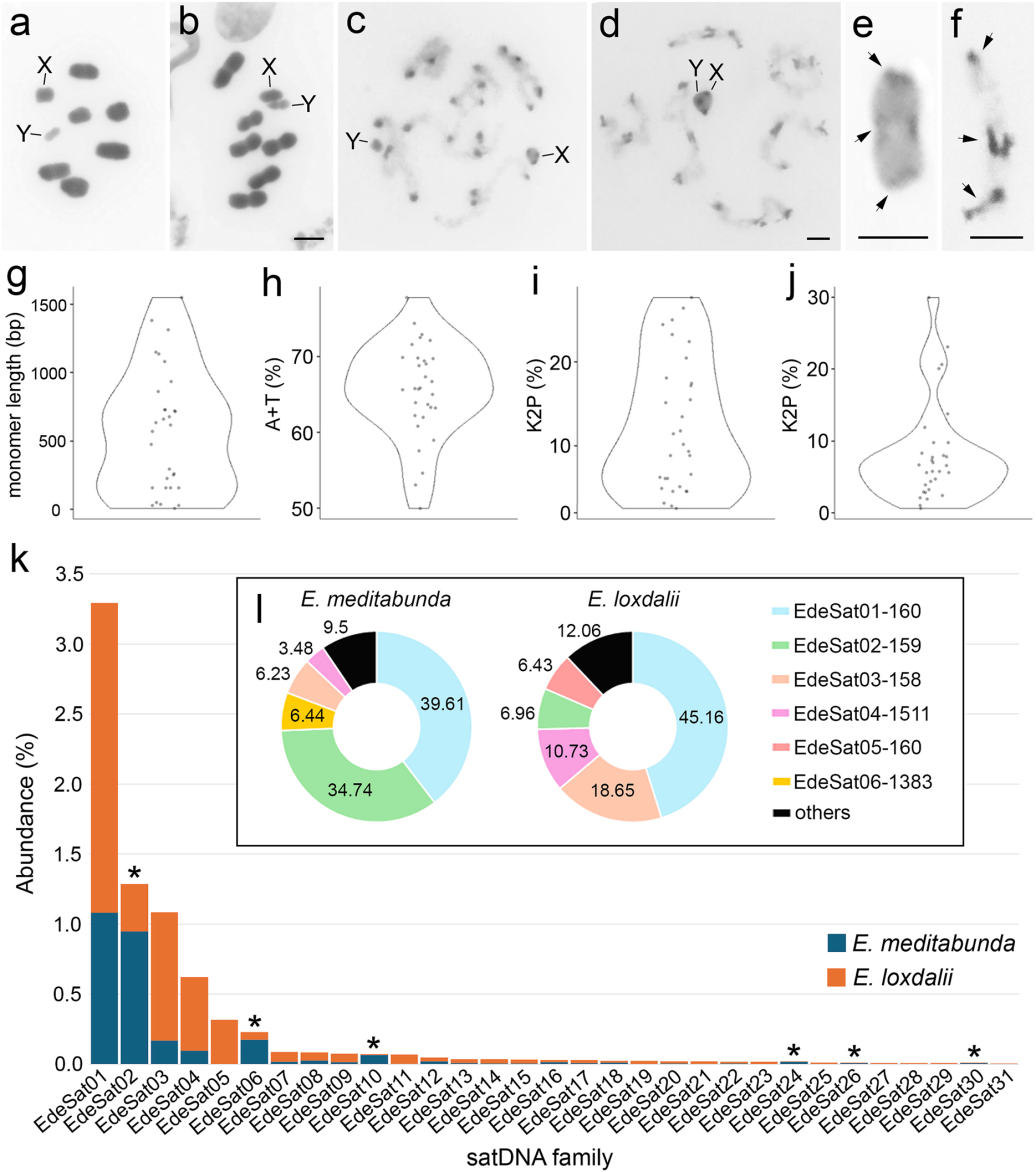
Chromosomes, heterochromatin distribution, and satellitome features of *Edessa meditabunda* and *Edessa loxdalii*. Metaphase I plates of *E. meditabunda* (**a**) and *E. loxdalii* (**b**) show six autosomal bivalents plus the sex chromosomes (2n = 14, XY). Heterochromatin distribution revealed by C-banding is shown in diplotene cells of *E. meditabunda* (**c**) and *E. loxdalii* (**d**). Insets highlight a single autosomal bivalent (**e**) and a diplotene bivalent (**f**) of *E. loxdalii*, evidencing C-positive heterochromatic blocks at both chromosomal termini (arrows). Panels (**g**–**j**) summarize general satellitome features: (**g**) monomer size distribution, with a predominance of families shorter than 1,000 bp; (**h**) A + T content, indicating enrichment above 60% in most monomers; (**i**, **j**) K2P divergence distributions for *E. meditabunda* and *E. loxdalii*, respectively, showing lower divergence (< 10%) for most satellite DNA (satDNA) families in *E. loxdalii* and a broader range of K2P in *E. meditabunda*. (**k**) Relative abundance of the 31 satDNA families in both species, with higher overall abundance in *E. loxdalii*. Asterisks indicate the only six satDNAs more abundant in the genome of *E. meditabunda*. (**l**) Relative contribution of the five most abundant satDNA families, four of which occur in the two species and together account for ~ 90% of each satellitome. Scale bar (**a**–**f**) = 5 µm

The karyotypes of both *Edessa* species consist of 2n = 14 chromosomes, with an XY sex chromosomes in males (Fig. 1a,b). Heterochromatin is present as conspicuous large blocks at both termini of the autosomes and the X chromosome, whereas the Y chromosome is entirely heterochromatic (Fig. 1c-f). This karyotype and heterochromatin pattern have been previously described for *E. meditabunda* (Rebagliati et al. 2005; Bardella et al. 2014), whereas for *E. loxdalii,* this is the first description.

Computational analysis identified 31 satDNA families present in the genomes of both *Edessa* species, and all characteristics of these families are detailed in Table 1. Sequence comparisons among the identified satDNAs revealed that they represent distinct families according to the classification criteria adopted in this study (homology ≥ 80%). The highest sequence similarity was observed between EdeSat01-160 and EdeSat05-160, corresponding to approximately 71%. Although this value is below the threshold used to define members of the same satDNA family, it may indicate a shared ancestral origin between these two families. None of the identified satDNAs met the criteria to be considered as sharing significant sequence homology with previously described repetitive elements (see methods). Although EdeSat03-158 falls within the threshold used for sequence comparison, it shares sequence similarity only with a TE identified in *Oryza sativa* (rice). Considering the large taxonomic distance between plants and insects, this match is unlikely to have biological significance and most likely represents a spurious sequence similarity rather than a shared evolutionary origin. The satDNAs presented nucleotide monomer lengths ranging from 10 bp to 1,551 bp (mean length of 575 bp), with most elements smaller than 1,000 bp (Fig. 1g). A predominance of A + T-rich sequences was observed (30 among the 31 satDNA families), with most satDNAs exhibiting more than 60% A + T content; only five showed values below this threshold (Fig. 1h). The analysis of read coverage across the trimer reference sequences supported the tandem organization of all 31 identified satDNAs in both species. Most satDNAs exhibited a relatively uniform coverage depth across the entire monomer and, importantly, across the junctions between adjacent monomers, consistent with the presence of sequencing reads spanning consecutive repeat units, as expected for tandemly organized repeats. Although non-uniform coverage was also noticed, including 14 satDNAs for *E. meditabunda* and four in *E. loxdalii*, these sequences can still be classified as satDNAs, as they retain evidence of tandemly repeated organization, even at lower copy number (Supplementary Fig. 1, Table 1).

**Table 1:** General features of the 31 satellite DNAs (satDNAs) identified in the male genomes of *Edessa meditabunda* and *Edessa loxdalii*. ML (monomer length in base pairs), AT (percentage of A + T content in monomers), K2P div (percentage of Kimura 2-parameter divergence), and GP (genomic proportion, %); Coverage: uniform = similar coverage along the nucleotide sequence and non-uniform = variability of coverage along the sequence. Note the overall enrichment in A + T content (mean > 65%), as well as the higher abundance and lower mean K2P divergence of satDNAs in *E. loxdalii* compared to *E. meditabunda*

			***E. meditabunda***			***E. loxdalii***		
satDNA family ID	ML	AT (%)	K2P div (%)	GP (%)	Coverage	K2P div (%)	GP (%)	Coverage
EdeSat01-160	160	65	11.83	1.079463	Uniform	3.33	2.212652	Uniform
EdeSat02-159	159	67.3	3.21	0.946578	Uniform	7.4	0.341228	Uniform
EdeSat03-158	158	63.3	8.83	0.169810	Uniform	23.13	0.913871	Uniform
EdeSat04-1511	1551	60.8	5.15	0.094782	Non-uniform	4.45	0.525714	Uniform
EdeSat05-160	160	69.4	20.5	0.000344	Non-uniform	2.46	0.315241	Uniform
EdeSat06-1383	1383	62	4.57	0.175580	Uniform	9.78	0.054989	Non-uniform
EdeSat07-935	935	74.4	25.06	0.018008	Non-uniform	11.92	0.069555	Uniform
EdeSat08-1316	1316	68.8	15.5	0.025738	Non-uniform	6.61	0.059318	Uniform
EdeSat09-619	619	72.5	11.49	0.015927	Non-uniform	7.07	0.057584	Uniform
EdeSat10-662	662	71.6	6.14	0.065854	Uniform	20.08	0.004815	Non-uniform
EdeSat11-1084	1084	77.8	27.74	0.001450	Non-uniform	5.8	0.068781	Uniform
EdeSat12-228	228	64	17.19	0.020091	Uniform	8.33	0.028912	Uniform
EdeSat13-1152	1152	62.2	4.94	0.009444	Uniform	2.88	0.026850	Uniform
EdeSat14-262	262	69.8	18.11	0.005365	Non-uniform	6.72	0.029706	Uniform
EdeSat15-255	255	69.8	17.48	0.005094	Uniform	5.84	0.027557	Uniform
EdeSat16-729	729	66.7	4.62	0.015043	Uniform	0.68	0.015419	Uniform
EdeSat17-1139	1139	63.7	6.25	0.007698	Uniform	1.08	0.020803	Uniform
EdeSat18-733	733	57.6	10.2	0.010948	Non-uniform	5.62	0.013298	Uniform
EdeSat19-722	722	69.9	13.53	0.001494	Non-uniform	1.93	0.021017	Uniform
EdeSat20-478	478	65.7	22.46	0.005596	Non-uniform	9.83	0.014400	Uniform
EdeSat21-53	53	54.7	24.52	0.002359	Uniform	13.83	0.016810	Uniform
EdeSat22-717	717	65.7	9.32	0.006613	Non-uniform	5.62	0.012116	Uniform
EdeSat23-296	296	65.9	15.19	0.000329	Non-uniform	7.85	0.016299	Uniform
EdeSat24-32	32	53.1	4.59	0.016192	Uniform	8.02	0.000003	Non-uniform
EdeSat25-39	39	59	6.64	0.003760	Uniform	7.77	0.007233	Uniform
EdeSat26-680	680	69	2.55	0.010043	Uniform	2.98	0.000013	Non-uniform
EdeSat27-865	865	65.8	23.36	0.000011	Non-uniform	3.92	0.008647	Uniform
EdeSat28-635	635	72.9	8.86	0.001969	Non-uniform	2.17	0.006314	Uniform
EdeSat29-30	30	63.3	6.11	0.000520	Uniform	4.72	0.007699	Uniform
EdeSat30-574	574	72.1	2.81	0.007918	Uniform	20.67	0.000079	Non-uniform
EdeSat31-10	10	50	26.52	0.001016	Uniform	29.96	0.002393	Uniform
Mean	575	65.6	12.43			8.14		
Total				2.725054			4.899334	

Statistical inferences indicated that the data for satDNA abundance and K2P divergence were non-normal (*p* < 0.05). Considering these attributes, correlations were assessed using Spearman’s rank coefficient (ρ). A significant positive correlation was detected for genome abundance (*ρ* = 0.415, *p* = 0.021), whereas K2P divergence showed no significant correlation (*ρ* = 0.165, *p* = 0.376).

The abundance and K2P divergence were highly variable among the distinct satDNA families at the intragenomic and interspecific levels. The general abundance of satDNAs was higher for *E. loxdalii,* representing 4.89% of its genome, while for *E. meditabunda,* the satDNAs represented 2.72% of the genome content. The mean K2P divergence was lower in *E. loxdalii* (8.14%), consistent with a higher amplification and homogenization of satDNAs in its genome compared to *E. meditabunda*, which showed a higher mean K2P (12.43%) and lower satDNA content. In *E. meditabunda* a K2P range of 2.55% to 27.74% was observed, with a greater proportion of satDNAs displaying higher divergence values than in *E. loxdalii* (Fig. 1i,j). The K2P values for *E. loxdalii*, ranged from 0.68% to 29.96%, with most satDNAs exhibiting K2P divergence below 10% (Fig. 1j). In contrast, the most abundant satDNA in the genomes of both *Edessa* was the same, EdeSat01-160, which represents about 1.08% of the *E. meditabunda* genome and 2.21% of the *E. loxdalii* genome. The least representative satDNA in the genome of *E. meditabunda* was EdeSat27-865 with 0.00001% of abundance and in *E. loxdalii* it was EdeSat24-32 with 0.000004%. These SatDNA families that are represented at extremely low genomic proportions should be interpreted cautiously, as estimates derived from low-coverage short-read data may not reliably reflect true genomic abundance or copy number near the detection limits of the approach. As expected, given the overall higher satDNA content, *E. loxdalii* exhibited a greater number of more abundant satDNA families, with 25 of the 31 identified satDNAs showing higher abundance in this species (Fig. 1k). Among the top five most abundant satDNAs in each species, four were coincident between the two species; although their relative rank positions differed, they included EdeSat01-160, EdeSat02-159, EdeSat03-158, and EdeSat04-1511. The EdeSat05-160 was another of the top five most abundant repeats in the genome of *E. loxdalii*, whereas in *E. meditabunda*, EdeSat06-138 was among the top five. Together, these satDNAs constitute the core satellitome, accounting for 90.50% of the satDNA content in *E. meditabunda* and 87.94% in *E. loxdalii* (Fig. 1l).

**Fig. 2:**
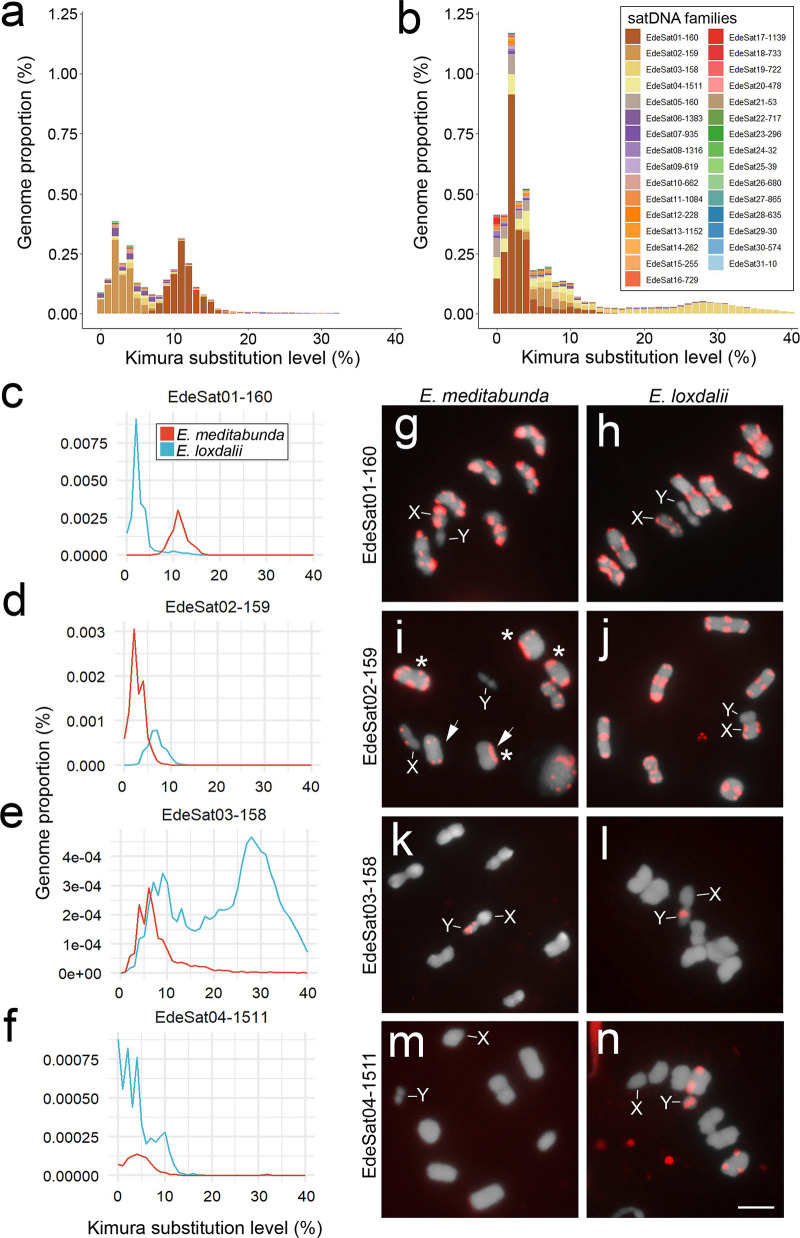
Genomic and chromosomal features of satellite DNAs (satDNAs) in *Edessa meditabunda* and *Edessa loxdalii*. (**a**, **b**) SatDNA landscapes (abundance *versus* divergence) for *E. meditabunda* and *E. loxdalii*, respectively, revealing patterns of amplification and homogenization. In *E. meditabunda*, two amplification peaks are evident, one for EdeSat01-160, corresponding to an older amplification, and one for EdeSat02-159, corresponding to a more recent amplification. In contrast, *E. loxdalii* shows a clear signature of more recent amplification for EdeSat01-160. Note the overall higher abundance of satDNAs in *E. loxdalii*, associated with lower K2P divergence values. (**c**–**f**) Individual landscapes of the four most abundant satDNAs (mean abundance across species), highlighting higher abundance peaks for three of them in *E. loxdalii* (EdeSat01-160, EdeSat03-159, and EdeSat04-1511). (**g**–**n**) Chromosomal mapping by FISH of four abundant satDNAs in *E. meditabunda* (g, i, k, m) and *E. loxdalii* (**h**, **j**, **l**, **n**). Probes are indicated in each panel. For EdeSat01-160 (**g**, **h**), signals span all heterochromatic blocks. For EdeSat02-159, in *E. meditabunda* (**i**) signals are restricted to one chromosomal terminus in two autosomes (arrows) and the X chromosome, but for other autosomes the signals are at both ends. Larger signals (asterisks) are observed in four autosomes, while the X chromosome and remaining two autosomes have smaller signals. In *E. loxdalii* signals are uniformly large and in both ends (**j**). EdeSat03-158 (**k**, **l**) is restricted to the Y chromosome in both species. EdeSat04-1511 (**m**, **n**) is detected only in *E. loxdalii*, on the Y chromosome and two autosomes. (**h**-**j**, **m**, **n**) metaphase I and (**g**, **k**, **l**) metaphase II. Scale bar (**g**–**n**) = 5 µm

The analysis of satDNA landscapes revealed divergent evolutionary dynamics between the two species, reflecting differences in the relative abundance and divergence of repeat elements, pointing to distinct patterns of amplification, turnover, and homogenization shaping their satellitomes. For *E. meditabunda,* two satDNA families showed peaks with considerable abundance: EdeSat01-160, with the highest abundance at a high K2P value, i.e., 11%, and EdeSat02-159, which had the highest abundance, about 0.30%, at a low K2P of 2%. EdeSat03-158 has a peak of abundance in K2P of 3–6%, and EdeSat04-1511 has a small peak of abundance around 1–7% (Fig. 2a,c–f). On the other hand, for *E. loxdalii* a dominant peak with high abundance (more than 0.80%) and with low K2P divergence (2%) is evident and dominated by EdeSat01-160. For EdeSat02-159, a small peak with K2P of 6–7% was evident. For EdeSat03-158, a double-peak abundance pattern was observed, one with high K2P divergence around 30% and another with K2P between 6–10%. For both EdeSat02-159 and EdeSat03-158, the peaks have much lower abundance than in EdeSat01-160. In EdeSat04-1511, the peak abundance was much higher than in *E. meditabunda,* with a peak between 0–9% K2P (Fig. 2b,c–f). Consistent with overall higher abundance and lower divergence for satDNA families in the genome of *E. loxdalii* in comparison to *E. meditabunda,* in this first species, some other satDNAs presented peaks of abundance in lower K2P. Only EdeSat06-1383, EdeSat10-662, EdeSat24-32, EdeSat26-680, and EdeSat30-574 show peaks with lower K2P in the *E. meditabunda* genome. Peaks of abundance in high K2P were observed for both species for EdeSat07-935, EdeSat12-228, and EdeSat31-10 (Supplementary Fig. 2).

Considering the mean abundance of satDNA families across species, we selected the four most abundant elements for FISH mapping on chromosomes, revealing clues about their chromosome organization and enrichment in terminal heterochromatin blocks. In both species, EdeSat01-160 occupied all heterochromatin blocks of the autosomes and the X chromosome (at both chromosome termini), with evidence of strong, conspicuous signals (Fig. 2g,h). The EdeSat02-159 was also placed on heterochromatic blocks of all autosomes and the X chromosome, but it presented differences in signal intensity at both intra- and interspecies levels. In the autosomes of *E. meditabunda,* the signals were much larger for the four large chromosomes, while in the two small autosomes and on the X chromosome, the signals were considerably smaller and with faint intensity. For three of the large and one small chromosome, the signals occurred on both chromosome termini. The X chromosome, one large and one small autosome had the EdeSat02-159 occupying only one chromosome terminus (Fig. 2i). For *E. loxdalii* no differences between chromosomes were observed, with all autosomes and X chromosomes presenting signals on both chromosome termini (Fig. 2j). For these two first satDNAs the Y chromosome did not present signals, although the EdeSat03-158 occurred exclusively in this chromosome of both species (Fig. 2k,l). The EdeSat04-1511 did not present clusters in the chromosomes of *E. meditabunda* (Fig. 2m), and for *E. loxdalii,* a distinctive pattern of chromosome distribution with signals on heterochromatin of two autosomes and on the Y chromosome was observed (Fig. 2n).

## Discussion

Satellitome analysis in Pentatomidae bugs is scarce, with description of satDNAs available only for *Euschistus heros* (Hickmann et al. 2023). The data from *E. heros,* along with our results for *Edessa*, although still limited to a few species, indicate that despite the conserved karyotype observed in pentatomids (2n = 14, XY), their genomes harbor dynamic satellitomes that, in the case of *Edessa,* are associated with extensive heterochromatin expansion. Reshuffling for repetitive DNAs in this group was also documented for other repetitive sequences, including TEs (Dionisio et al. 2023), multigene families (Bardella et al. 2016; Souza-Firmino et al. 2020), and heterochromatin base-pair enrichment composition (Bardella et al. 2013; Dionisio et al. 2023). The extensive amplification of heterochromatin in *Edessa* and conservation of diploid number (Bardella et al. 2013; Dionisio et al. 2020) also reinforce the idea that substantial genomic reorganization can occur without large changes on chromosomes. Occurrence of large heterochromatic blocks in *Edessa* resembles the structure of monocentric chromosomes in which there is a localized center that favors the repeats expansion, i.e., the centromere, and for Pentatomidae is a particular case, as almost all other species have low heterochromatin content (Bardella et al. 2013; Dionisio et al. 2020). Besides the two *Edessa* studied here, large blocks of heterochromatin were observed in the other three species, *Edessa rufomarginata*,* Edessa impura*, and *Edessa collaris* (Bardella et al. 2013; Dionisio et al. 2020), suggesting that it can be a common placement in the genus, but only with analysis of other species can this idea be confirmed. In other insects with holocentric chromosomes, including Hemiptera and Lepidoptera, large heterochromatic blocks are also exceptional. As observed in *Edessa*, these rare cases are associated with satDNA expansion (Pita et al. 2017; Bardella et al. 2020; Mora et al. 2023; Cabral-de-Mello et al. 2024; Gasparotto et al. 2026), whereas most species studied so far exhibit relatively low heterochromatin content. The only sporadic occurrence of large amounts of heterochromatin in these insects could be explained by the fact that the formation of long arrays of tandem repeats requires a specific genomic environment, which is likely limited in holocentric chromosomes, as the located centromere is absent.

Although the genomes of *Edessa* contain a lower overall proportion of satDNAs than *E. heros* (approximately 4.4% of the genome; Hickmann et al. 2023), the most abundant satDNA families in *Edessa* individually account for a larger fraction of the genome than the dominant satDNA families in *E. heros*. This pattern suggests that amplification in *Edessa* has been concentrated in a few satDNA families. In this way, the involvement of satDNAs in the expansion of heterochromatin in *Edessa* is suggested, as we clearly documented by chromosomal mapping of the four most abundant elements, some of which were enriched in these areas, forming conspicuous blocks. As mentioned before, heterochromatin expansion driven by satDNAs was documented in other Hemiptera, including the cases of *Holhymenia histrio*, Coreidae (Bardella et al. 2020), and *Triatoma delpontei*, Reduviidae (Mora et al. 2023), which has the largest amount of satDNA described up to now, i.e., more than 50% of the genome. In monocentric insects, striking case occurs in *Melipona scutellaris* bee, in which one satDNA accounts for 38.2% of the genome and occupies the entire heterochromatin of the species (Pereira et al. 2021). Also, in the beetle *Tenebrio molitor,* high heterochromatin content, with a predominance of satDNA (up to 50% of the genome), was observed (Juan and Petitpierre 1989; Mora et al. 2024). Although in *Edessa* the heterochromatin formed large blocks, the satDNAs showed more restricted expansion, occupying a much smaller portion of the genome than the cited examples. Especially the EdeSat01-160, which is the most abundant satDNA in the genomes of *E. meditabunda* and *E. loxdalii,* has the same chromosomal distribution in heterochromatin of both species, evidencing partial conservation of these regions. A similar pattern is observed for EdeSat02-159, as this satDNA also constitutes part of the heterochromatin of autosomes and the X chromosome, but differences in signal intensity and location were noted, revealing greater amplification of this repeat in one species, i.e., *E. meditabunda*, and its involvement in reshaping the current heterochromatin landscape between species. Interestingly, the amplification of EdeSat02-159 in *E. meditabunda* occurred primarily on the large autosomes, reshaping the satDNA heterochromatin landscape at the intragenomic level and highlighting chromosome-specific patterns of repeat accumulation resulting from local amplification. Local amplification of a satDNA on larger chromosomes was also noticed for HisSat01-184 in the Coreidae *H. histrio* (Bardella et al. 2020).

The more restricted chromosomal distribution of EdeSat03-158 and EdeSat04-1511 indicates that these families contributed less to the overall heterochromatin landscape, whereas the most abundant satDNAs, which occupy a larger number of chromosomes and heterochromatic blocks, were the main contributors to heterochromatin expansion. EdeSat03-158 is limited to a large Y-chromosome block in both species, while EdeSat04-1511 occurs on the Y and two autosomes in *E. loxdalii* but shows no signals in *E. meditabunda*, consistent with its reduced coverage along the sequence in this species, revealing a lower tandem pattern hampering FISH signal observation. Like for EdeSat02-159, the differential distribution of EdeSat04-1551 on autosomes of the two *Edessa* supports satDNA heterochromatin landscape differentiation at the intragenomic and interchromosomal levels. Concerning the Y chromosome, the differential amplification of repeats and the specific satDNA landscape in regards to other chromosomes observed for this element are expected, as this chromosome has restricted recombination, favoring the plasticity and erosion of this element by action of mechanisms such as unequal sister chromatid exchange, replication slippage, and TE-mediated dispersal, which together promote the expansion and turnover of satDNA arrays (Dover 1982; Charlesworth et al. 1994; Hobza et al. 2015; Garrido-Ramos 2017). Moreover, the absence of some of the most abundant satDNAs, such as EdeSat01-160 and EdeSat02-159, suggests differential repeat dynamics in this chromosome, indicating its particular evolution in comparison to autosomes. Differential accumulation of satDNAs and repeat landscapes between sex chromosomes, involved in their evolution, was documented in multiple other species of insects, as in the beetle *Omophoita octoguttata* (Vidal et al. 2025a), the cricket *Eneoptera surinamensis* (Palacios-Gimenez et al. 2017), the grasshopper *Ronderosia bergii* (Ferretti et al. 2020), and in some Triatominae species (Panzera et al. 2023), as well as in other animals (de Oliveira et al. 2024; Aleix-Mata et al. 2025; Sassi et al. 2025; Vidal et al. 2025b) and plants (Puterova et al. 2017; Hobza et al. 2017). The conserved occurrence of EdeSat03-158 on the Y chromosome may initially appear unexpected, given the high dynamism of satDNA and of the Y chromosome. From an evolutionary perspective, this pattern may reflect the retention of ancestral satDNA families, suggesting that these sequences were already associated with the Y chromosome in the common ancestor and have been maintained despite the rapid turnover typically observed for this chromosome. Conservation of satDNAs in the heterochromatic Y chromosome with high abundance in multiple species was documented for GATA repeat in Triatomini, suggesting an ancestral trait (Panzera et al. 2023). Although EdeSat03-158 is conserved on the Y chromosome in both species, it shows clear evidence of evolutionary differentiation, as indicated by highly divergent K2P values and distinct amplification patterns. In *E. loxdalii*, this satDNA exhibits two waves of amplification, an older event followed by a more recent one, whereas in *E. meditabunda*, amplification appears to have occurred only more recently. Further differentiation of the Y chromosome between the species is supported by EdeSat04-1511, which is present on the Y chromosome of *E. loxdalii* but absent in *E. meditabunda*.

Besides chromosomal distribution, satellitome analyses reveal contrasting molecular evolutionary patterns between the two *Edessa* species. *Edessa loxdalii* exhibits higher satDNA abundance and lower K2P divergence, suggesting recent amplification and homogenization, whereas *E. meditabunda* shows lower abundance and higher divergence, consistent with more advanced sequence degeneration. These differences are exemplified by low-divergence peaks in *E. loxdalii* for EdeSat01-160, indicative of recent expansion, while *E. meditabunda* presents peaks at higher divergence values and with lower abundance. An opposite pattern is observed for EdeSat02-159, which is more abundant and homogeneous in *E. meditabunda*. The presence of multiple divergence peaks within satDNA families supports successive waves of amplification following species divergence. Although there are differences in abundance for some satDNAs between species, a moderate but significant correlation in satDNA abundance occurs, indicating the quantitative maintenance of a shared satellitome repertoire inherited from a common ancestor. In contrast, the lack of correlation in sequence divergence supports independent evolutionary trajectories, reflecting lineage-specific differential homogenization. These data are consistent with the library hypothesis (Fry and Salser 1977). The occurrence of highly homologous satDNA families with distinct abundances (EdeSat01-160 and EdeSat05-160) supports the diversification of distinct satDNA families through sequence divergence while retaining detectable homology, in which ancestral satDNA variants diversify and are differentially amplified during genome evolution. The skewed abundance distribution further reflects a typical satellitome structure, with few highly amplified families and many low-copy repeats. Together, these results support a model of concerted evolution evidenced in some groups (e.g. Utsunomia et al. 2017; Palacios-Gimenez et al. 2020; de Lima and Ruiz-Ruano 2022; Gutiérrez et al. 2022; Peona et al. 2023; Sales-Oliveira et al. 2024; Senderowicz et al. 2025), in which shared satDNA families are differentially amplified across lineages despite their common origin (Dover 1982; Camacho et al. 2022).

The analysis of satDNA dynamics on holocentric chromosomes remains relevant for understanding genome architecture, as satDNAs are not coupled with localized centromere function. In this way, our study expands knowledge of satDNA dynamics in insects with holocentric chromosomes, particularly in species with relatively high heterochromatin content compared with most other holocentric insects, such as the *Edessa* representatives studied here. Our results demonstrate that satDNA dynamics play a role in shaping genome organization in *Edessa*, as they are involved with heterochromatin expansion despite an overall conserved karyotypic framework and no remarkable expansion of satellitome. Moreover, we noticed marked quantitative differences in satellitome composition and evolutionary dynamics, as well as in the reshaping of heterochromatic blocks and the Y chromosome. It highlights the role of differential repeat dynamics in promoting genomic diversification between the two species, despite the conservation of the satDNA core, as most abundant satDNA families dominate the satellites of both species, and heterochromatic blocks show partial composition conservation. Analysis of other *Edessa* species will be valuable for addressing open questions, such as the possible ancient amplification of heterochromatin in the genus and how satDNAs evolve at phylogenetic context within unusually large heterochromatin blocks on holocentric chromosomes. Moreover, analysis of other repetitive classes, such as TEs, could also provide information on patterns of heterochromatin amplification and differentiation in the genus.

## Supplementary Information

Below is the link to the electronic supplementary material.

Supplementary file1: Supplementary Figure 1. Examples of satellite DNA profiles for the four most abundant families in the genomes of *Edessa meditabunda* and *Edessa loxdalii*. Coverage and nucleotide variability are shown for each position along the trimer consensus sequences. The first three families exhibit a typical coverage pattern expected for tandemly arrayed sequences, i.e., relatively uniform coverage across the sequence. In contrast, EdeSat04-1511 shows a clear decrease in coverage in *E. meditabunda*, suggesting that its copies are not organized only as tandem arrays. The arrows indicate regions of nucleotide sequence with lower coverage. (DOCX 337 kb)

Supplementary file2: Supplementary Figure 2. Individual landscapes (abundance versus divergence) for the satellite DNAs identified in the genomes of *Edessa meditabunda* and *Edessa loxdalii*. (DOCX 661 kb)

Supplementary file3: Supplementary Table 1. List of primers used for amplification of four satellite DNAs (satDNAs) mapped on chromosomes of *Edessa meditabunda* and *Edessa loxdalii*. (DOCX 14 kb)

## Data Availability

No datasets were generated or analysed during the current study.
