# Supplementary figures and images for: Satellite DNA evolution in two holocentric species of Edessa true bugs (Hemiptera: Pentatomidae) with unusually high heterochromatin abundance
Source: Chromosome Res. 2026 Jul 31;34(1):17. doi: 10.1007/s10577-026-09809-2 (PMC13427858; doi:10.1007/s10577-026-09809-2)

**Supplementary Figure 1.**

**
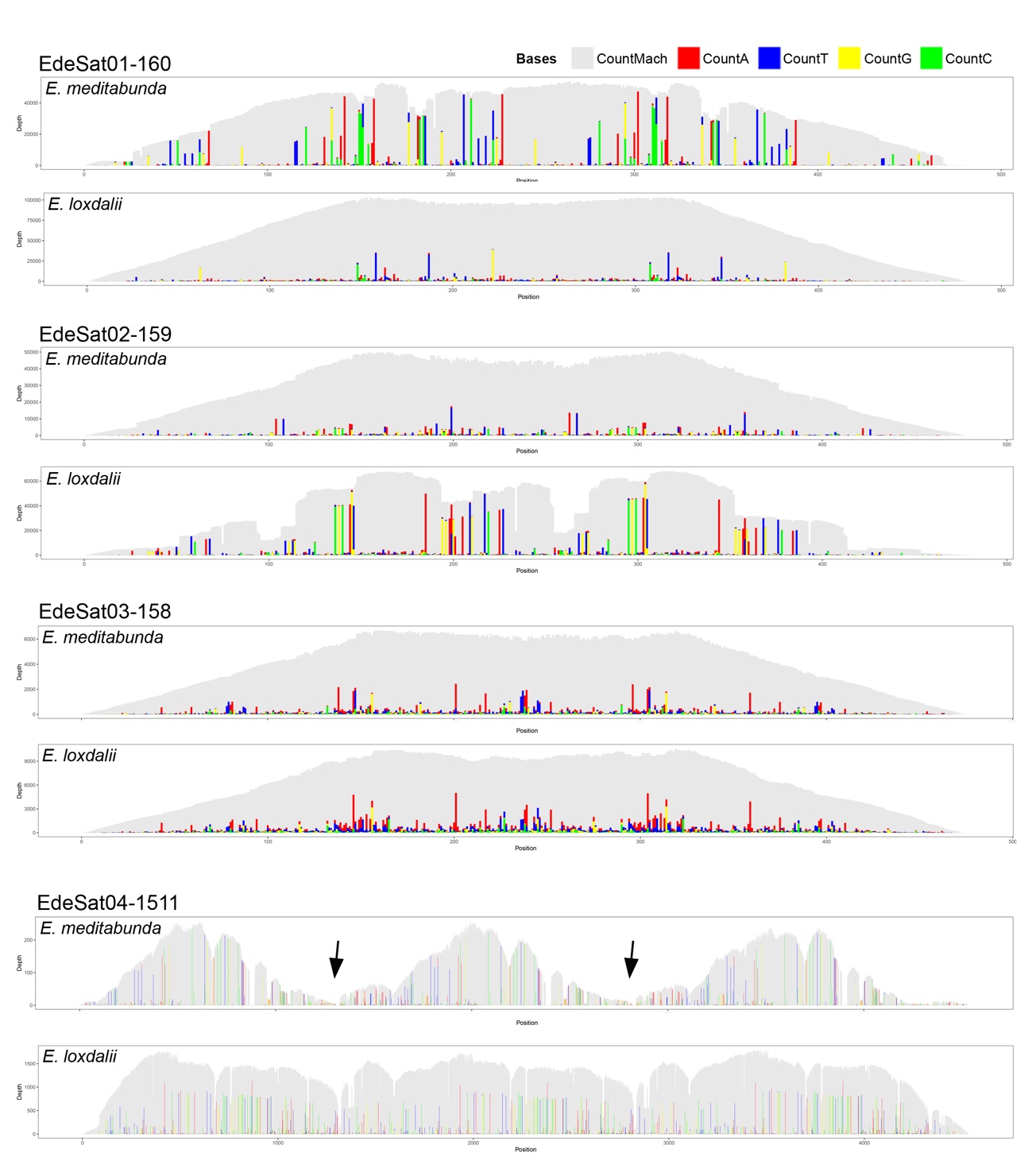
**

Supplement: Supplementary file 1 — Supplementary file1: Supplementary Figure 1. Examples of satellite DNA profiles for the four most abundant families in the genomes of Edessa meditabunda and Edessa loxdalii. Coverage and nucleotide variability are shown for each position along the trimer consensus sequences. The first three families exhibit a typical coverage pattern expected for tandemly arrayed sequences, i.e., relatively uniform coverage across the sequence. In contrast, EdeSat04-1511 shows a clear decrease in coverage in E. meditabunda, suggesting that its copies are not organized only as tandem arrays. The arrows indicate regions of nucleotide sequence with lower coverage. (DOCX 337 kb) [file 10577_2026_9809_MOESM1_ESM.docx]

**Supplementary Figure 2.**

**
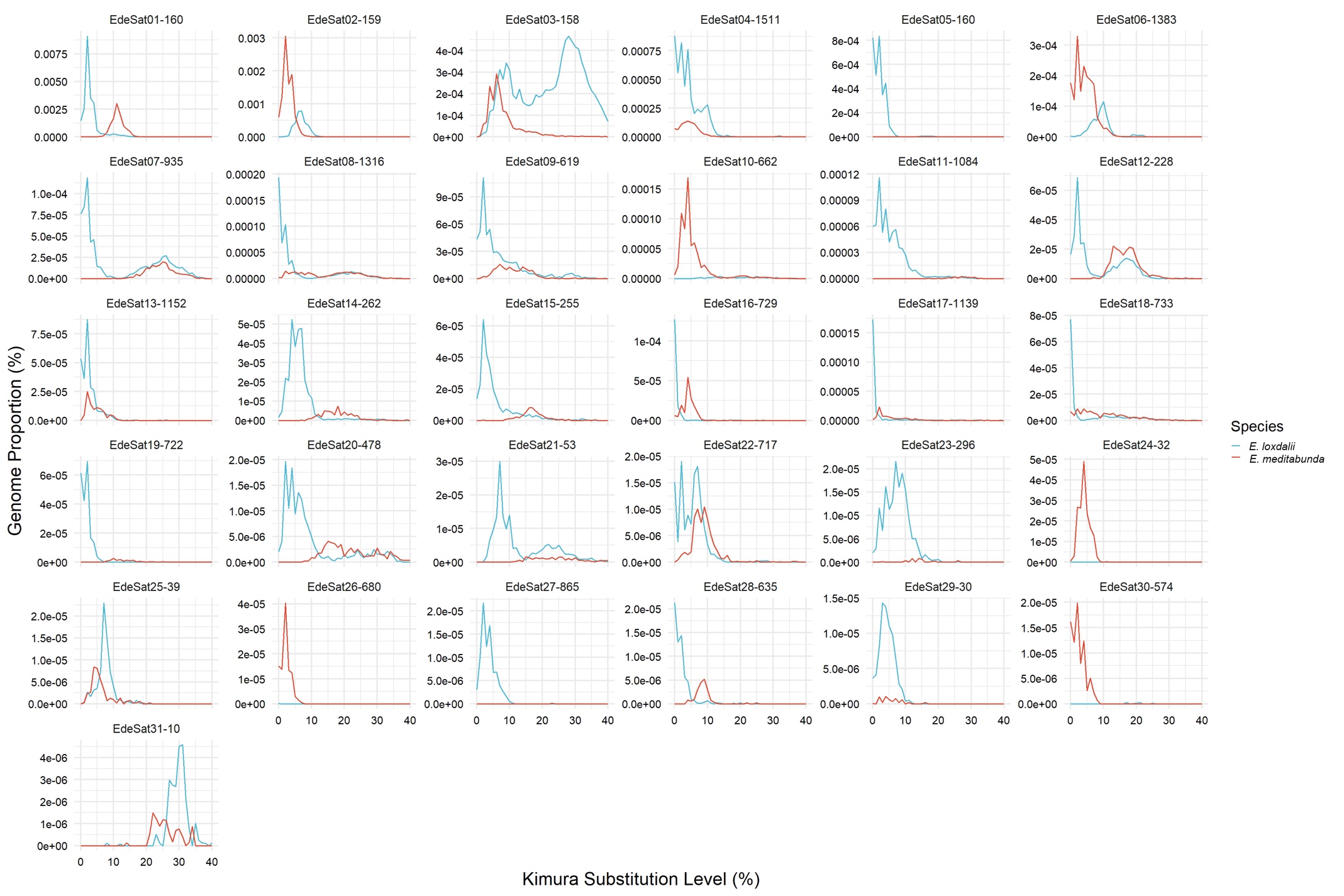
**

Supplement: Supplementary file 2 — Supplementary file2: Supplementary Figure 2. Individual landscapes (abundance versus divergence) for the satellite DNAs identified in the genomes of Edessa meditabunda and Edessa loxdalii. (DOCX 661 kb) [file 10577_2026_9809_MOESM2_ESM.docx]
