# Supplementary material for: Satellite DNA evolution in two holocentric species of Edessa true bugs (Hemiptera: Pentatomidae) with unusually high heterochromatin abundance
Source: Chromosome Res. 2026 Jul 31;34(1):17. doi: 10.1007/s10577-026-09809-2 (PMC13427858; doi:10.1007/s10577-026-09809-2)
Supplement: Supplementary file 3 — Supplementary file3: Supplementary Table 1. List of primers used for amplification of four satellite DNAs (satDNAs) mapped on chromosomes of Edessa meditabunda and Edessa loxdalii. (DOCX 14 kb) [file 10577_2026_9809_MOESM3_ESM.docx]

**Supplementary Table 1.**

| **SatDNA ID** | **F** | **R** |
| --- | --- | --- |
| EdeSat01-160 | 5’ TCATACTAGCGCTGGATTACA | 5’ CGATCCAAAAGAAAAAGTCGC |
| EdeSat02-159 | 5’ GTAATTTGATGTGGTATCGAC | 5’ ATTAGAAACGCGAATCAGACA |
| EdeSat03-158 | 5’ CCAACACTAGGTTCCGGTTT | 5’ GCACCTCCCCCTCTTTATTAT |
| EdeSat04-1511 | 5’ GAGGTATACCTCTACTGGTG | 5’ TACACTCCACTACAGTACGC |
